# Predictive Factors for the Common Adverse Maternal and Fetal Outcomes in Pregnancies Complicated by Systemic Lupus Erythematosus

**DOI:** 10.1089/whr.2023.0180

**Published:** 2024-05-17

**Authors:** Qianwen Dai, Mengtao Li, Xinping Tian, Yijun Song, Jiuliang Zhao

**Affiliations:** ^1^ Department of Obstetrics and Gynecology, Peking Union Medical College Hospital, Beijing, China.; ^2^ Dept of Rheumatology, Peking Union Medical College Hospital, Peking Union Medical College and Chinese Academy of Medical Sciences, National Clinical Research Center for Dermatologic and Immunologic Diseases (NCRC-DID), Key Laboratory of Rheumatology and Clinical Immunology, Ministry of Education, Beijing, China.

**Keywords:** systemic lupus erythematosus, pregnancy, adverse pregnancy outcomes, preterm birth, preeclampsia, predictors

## Abstract

**Objectives:**

This study aimed to evaluate the outcomes of pregnancy in patients with systemic lupus erythematosus (SLE). It focused on identifying clinical and laboratory markers that could predict the common adverse pregnancy outcomes (APOs) after 20 weeks of gestation, namely preeclampsia (PE) and preterm birth (PTB) in them.

**Methods:**

Pregnant SLE women who delivered at the study center from 2010 to 2023 were retrospectively analyzed. Categorical variables were evaluated using the chi-square test or Fisher’s exact test, while continuous variables underwent Mann–Whitney *U* testing. Stepwise regression was used to assess the predictors of pregnancy outcomes.

**Results:**

The study enrolled 445 pregnancies in 408 women diagnosed with SLE. Of these, 202 pregnancies (45.4%) resulted in at least one APO. Disease flare-ups, hypertension, and proteinuria during the first trimester were primary predictors of at least one APO and PTB. The most frequently recorded maternal adverse outcome was PE (14.6%), while PTB accounted for 32.6% of fetal adverse outcomes. Multivariate regression analysis identified hypertension, history of PE, associated antiphospholipid syndrome (APS), proteinuria, and low serum C4 in the first trimester as independent risk factors for PE. Regular follow-ups at our center correlated with lower risks of APOs, PE, and PTB. APS also emerged as a risk factor for PTB, whereas the use of hydroxychloroquine (HCQ) during pregnancy seemed to protect against PTB.

**Conclusion:**

For pregnancies complicated by SLE, we recommend early pregnancy screening for proteinuria—even in the absence of lupus nephritis—as well as continued use of HCQ and routine prenatal care throughout pregnancy.

## Introduction

Systemic lupus erythematosus (SLE), a chronic, multisystem, connective tissue autoimmune disease, predominantly affects women of reproductive age. Although fertility typically remains unaffected in SLE patients who do not undergo cytotoxic treatment, concerns regarding the health of both the mother and fetus during pregnancy persist.^1–3^ Pregnancies in SLE patients tend to have higher rates of adverse pregnancy outcomes (APOs), such as preterm birth (PTB), preeclampsia (PE), and fetal loss, compared to those in healthy women.^4,5^

Significant strides have been made in improving the pregnancy outcomes of SLE patients, thanks to careful planning of conception and comprehensive health care provided during periods of disease inactivity. However, it is crucial to acknowledge that pregnancies in SLE patients still bear a heightened risk of APOs. Prior studies have identified several risk factors, including active disease, hypocomplementemia, presence of anti-double stranded deoxyribonucleic acid (anti-dsDNA) antibodies, antiphospholipid antibodies (aPLs), lupus nephritis (LN), and maternal hypertension (HTN), associated with an increased probability of experiencing APOs.^5–7^ Buyon and colleagues carried out a prospective multicenter study involving 385 patients, aiming to explore potential predictors of APOs in patients with inactive or stable SLE. Remarkably, APOs affected nearly 20% of the patients under investigation. Several baseline predictors for APOs were identified, among which the presence of lupus anticoagulant (LA) and usage of antihypertensive drugs stood out as the strongest. They were associated with an elevated APO rate of 58.0%, along with a neonatal mortality rate of 22.0%.^8^

Despite extensive and ever-growing research, we lack a clear understanding of the relationship between SLE and pregnancy. It is imperative to determine the association between SLE and APOs, particularly PE and PTB, which significantly contribute to prenatal morbidity and mortality rates. This understanding will be critical for health care providers who aim to improve pregnancy outcomes.

Consequently, we conducted a retrospective study of pregnant women with SLE who delivered at our center, with the goal of identifying clinical and laboratory indicators in the first trimester that can predict APOs, especially PE and PTB. By doing so, we aim to provide ample guidance for implementing innovative therapies in the future.

## Methods

### Ethical approval

The need for informed consent was exempted due to the retrospective and observational design of the study, with the approval of the ethics committee (K4483). All procedures conducted in this study adhered to the guidelines outlined in the Declaration of Helsinki and its revisions or equivalent ethical standards.

### Study design

A retrospective observational study was conducted at the study center from January 2010 to May 2023 by retrieving complete medical records of women who were diagnosed with SLE before or during pregnancy. These SLE patients met at least four of 11 criteria of the revised American College of Rheumatology 1997 classification criteria or the 2012 Systemic Lupus International Collaboration Clinic Classification criteria.^9,10^ Patients with other autoimmune diseases [except associated antiphospholipid syndrome (APS)] were not included. Patients who had spontaneous abortions, artificial abortions before 20 weeks of gestation, or twin pregnancy were also excluded.

### Variables of interest

Baseline patient characteristics included age at conception, method of conception, antenatal clinic, parity, duration of SLE, history of thrombosis, history of PE, HTN, LN, diabetes mellitus (DM), previous APOs, and previous manifestations of SLE. We also collected disease flare-ups and medications during pregnancy. According to the Definition of Remission in SLE (DORIS) task force and 2022 Chinese guideline for the management of pregnancy and reproduction in patients with systemic lupus erythematous, in our study, “remission” refers to the absence of active disease manifestations, including clinical symptoms and laboratory abnormalities, for at least six months prior to conception, irrespective of serology. The patients may be on antimalarial, low-dose glucocorticoids (prednisolone ≤ 15 mg daily) and/or stable immunosuppressive drugs, including biologics.^11,12^ Disease activity was scored by the SLE Disease Activity Index-2000 (SLEDAI-2K) adapted to pregnancy.^13,14^ A total score of >4, new onset of SLE during pregnancy, the addition of immunosuppressive medications or hydroxychloroquine (HCQ), or an increase in prednisone ≥0.5 mg/kg/day were defined as a disease flare. For cases of LN where a conclusive diagnosis could not be established through renal biopsy, we identified nephritis based on the presence of proteinuria (≥0.5 g/day) or the presence of cellular casts. Women at our center were seen at least once a month up to the 28th week of gestation, every two weeks from the 28th week up to the 36th week, and then every week until delivery by a multidisciplinary team that included experienced obstetricians and rheumatologists.

Laboratory values included anti-dsDNA antibodies, anti-SSA antibodies, anti-SSB antibodies, complement 3 (C3), complement 4 (C4), and aPLs. aPL status included anticardiolipin (aCL), anti-Beta2 glycoprotein type I (anti-β2GPI) antibodies, and LA. To search for predictors of APOs, all the laboratory variables were collected during the first trimester.

### Definitions of adverse pregnancy outcomes

Maternal outcomes included type of delivery, gestational age at delivery, and obstetric outcomes. Adverse maternal outcomes included PE, preterm premature rupture of membrane (pPROM), and maternal death.

PE was defined as the new onset of hypertension (blood pressure ≥140/90 mmHg) after 20 weeks of gestation with either proteinuria (≥0.3 g/24 h) or any other organ dysfunction, including kidney, liver, and placental dysfunction. For the diagnosis of eclampsia, seizures had to be present and other causes had to be ruled out.^15^

pPROM was defined as the spontaneous premature rupture of membranes before 37 weeks, and maternal mortality was defined as the death of a woman due to complications from pregnancy, childbirth, or within 42 days after termination.^16,17^

Adverse fetal outcomes included PTB, stillbirth, small for gestational age (SGA), neonatal death, and 1-minute Apgar score <7.

According to ACOG, PTB was defined as a delivery occurring at or after 20 0/7 weeks of gestation and before 37 0/7 weeks of gestation.^18^

Stillbirth was defined as fetal death that occurs during pregnancy ≥20 weeks, and neonatal death referred to the death of a newborn baby within 28 days of birth.^19^

SGA was used to describe newborns whose birth weight was less than the 10th percentile for gestational age.^20^

APOs were any of the above adverse maternal and fetal outcomes.

### Statistics

All analyses were carried out using IBM SPSS Statistics 26. Continuous variables are presented as means with standard deviations (SDs) for normally distributed data and medians with interquartile ranges (IQRs) for non-normally distributed data. Categorical variables are represented by frequencies and percentages. The chi-square test or Fisher’s exact test was used to compare categorical variables, whereas the Mann–Whitney *U* test was used for continuous variables given the non-normal distribution of pregnancies with and without APOs. A subanalysis was also conducted for each maternal and fetal complication, namely, PE and PTB.

Receiver operating characteristic (ROC) analysis was performed to calculate the critical cutoff values of C3, C4, and 24-hour proteinuria for predicting APOs, PE, and PTB. Variables that showed statistical significance (*p* < 0.05) in the univariate analysis or those deemed clinically relevant were further analyzed as predictors of APOs/PE/PTB using stepwise regression. Each pregnancy was considered an individual observation. In all cases, a two-sided *p* value < 0.05 was considered statistically significant.

## Results

### Patient characteristics

The study encompassed 408 SLE patients accounting for a total of 445 pregnancies. The majority of these patients had one pregnancy in the study period (91.7%), while 31 patients had two pregnancies and another three had three pregnancies. Over 96% of the pregnancies were naturally conceived, and 82.2% of the pregnancies were in primiparous women. Forty-six (10.3%) patients were diagnosed with SLE during their pregnancy, which was defined as new-onset SLE.

Table 1 presents a comprehensive view of maternal background characteristics and medications. The median age of conception was 31 years, with a median duration of SLE of 5 years. Of the total, 345 (77.5%) patients received prenatal care and came to regular follow-up at our center. More than 94% of the pregnancies were planned, with disease remission for over six months preceding conception. A total of 105 (23.6%) patients experienced a disease flare-up during pregnancy. Notably, LN was reported in 172 (38.7%) pregnancies, and 42 (9.4%) cases featured secondary APS. When SLE was diagnosed, mucocutaneous lesions were the most frequently observed clinical manifestation, occurring in 42.5% of cases. Arthritis followed closely, being found in 35.1% of all pregnancies.

**Table 1.** Maternal Background Characteristics and Medications in SLE Pregnancies

Variables	Total (*n* = 445)
Maternal characteristics	
Assisted reproductive technology, *n* (%)	14 (3.1)
Age at conception, Median (Q1, Q3)	31 (28, 33)
Regular follow-up, *n* (%)	345 (77.5)
SLE duration (years), Median (Q1, Q3)	5.11 (2.81, 9)
Nulliparity, *n* (%)	366 (82.2)
Planned pregnancy, *n* (%)	378 (94.7)
HTN, *n* (%)	19 (4.3)
LN, *n* (%)	172 (38.7)
Associated APS, *n* (%)	42 (9.4)
History of PE, *n* (%)	16 (3.6)
Previous thrombosis, *n* (%)	14 (3.1)
History of APOs, *n* (%)	121 (27.2)
Treatment during pregnancy	
LDA, *n* (%)	138 (31)
Prednisone, *n* (%)	350 (78.7)
LMWH, *n* (%)	50 (11.2)
HCQ, *n* (%)	357 (80.2)
Immunosuppressive drug, *n* (%)	94 (21.1)
Manifestations of SLE prepregnancy	
Rash, *n* (%)	189 (42.5)
Blood system, *n* (%)	156 (35.1)
Nervous system, *n* (%)	17 (3.8)
Arthritis, *n* (%)	161 (36.2)
Low platelet count, *n* (%)	113 (25.4)
Urinary sediment, *n* (%)	138 (31)
Laboratory characteristics in the first trimester	
24-h proteinuria (g/L), Median (Q1, Q3)	0.1 (0.08, 0.16)
Positive anti-dsDNA antibodies, *n* (%)	138 (31)
Positive anti-SSA antibodies, *n* (%)	225 (50.6)
Positive anti-SSB antibodies, *n* (%)	57 (12.8)
At least one positive aPL, *n* (%)	104 (23.4)
Complement C3, Mean ± SD	0.88 ± 0.24
Complement C4, Median (Q1, Q3)	0.14 (0.11, 0.17)
Pregnancy outcomes	
Gestation weeks, Median (Q1, Q3)	38 (35.4, 38.5)
Cesarean section, *n* (%)	304 (68.3)
Disease flare-ups, *n* (%)	105 (23.6)
Birth weight, g, Median (Q1, Q3)	2820 (2435, 3135)
Fetal sex, *n* (%)	
Boy	225 (51.1)
Girl	215 (48.9)

LN, lupus nephritis; HTN, hypertension; PE, preeclampsia; APOs, adverse pregnancy outcomes; PTB, preterm birth; SLE, systemic lupus erythematosus; APS, antiphospholipid syndrome; LDA, low-dose aspirin; LMWH, low molecular weight heparin; HCQ, hydroxychloroquine; Immunosuppressive drugs, such as azathioprine (AZA), tacrolimus (TAC), and intravenous immunoglobulin (IVIG); anti-dsDNA, anti-double stranded DNA; aPL, antiphospholipid; SGA, small for gestational age.

Of all the pregnancies, 19 (4.3%) were associated with HTN, 78.9% of which resulted in APOs. Only one patient had progestational diabetes mellitus (not shown in Table 1), and 16 (3.6%) had a history of PE. One hundred and twenty-one (27.2%) had a history of APOs, and 14 (3.1%) had a history of thrombosis.

Regarding medications initiated in early pregnancy, only 138 (31.0%) pregnancies involved the use of aspirin. Hydroxychloroquine (HCQ) was taken in nearly 357 (80.2%) pregnancies, while prednisone was used in 350 (78.7%) pregnancies. Low-molecular-weight heparin (LMWH) was used in 50 (11.2%) pregnancies, and immunosuppressive drugs were prescribed in 94 (21.1%). All medication decisions were made at the discretion of the treating physician.

### Adverse pregnancy outcomes

Table 2 summarizes the obstetric outcomes. The average gestational age at delivery was 38 weeks, and the average birth weight was 2820 g. Of the 455 pregnancies, nearly 70% were terminated by cesarean section for various reasons, which included social background or obstetrical complications. At least one APO occurred in 202 pregnancies (45.4%): 30 (6.7%) pPROM, 145 (32.6%) PTB, 65 (14.6%) PE, and one case of eclampsia during pregnancy. Within the cohort of 408 SLE patients, three women died from active disease, specifically pulmonary arterial hypertension (PAH), cardiomyopathy, or concurrent infection. Regarding the outcomes of the fetuses and newborns, there were 19 (4.3%) stillbirths, 113 (25.4%) SGA, and 14 (3.1%) newborns who did not survive. The main cause of neonate death was prematurity, and 9 (64.3%) of them were not resuscitated due to being identified as having a young gestational age, as their families decided against intervention. Finally, 9 (2.0%) of the newborns had a 1-minute Apgar score of less than 7.

**Table 2.** Obstetric Outcomes

Obstetric outcomes	Total pregnancies (*n* = 445)
Gestational age at delivery, w (Q1, Q3)	38 (35.4, 38.5)
Birth weight, g (Q1, Q3)	2820 (2435, 3135)
At least one APO	202 (45.4)
pPROM, *n* (%)	30 (6.7)
PTB, *n* (%)	145 (32.6)
PE, *n* (%)	65 (14.6)
Maternal death, *n* (%)	3 (0.7%)
Stillbirth, *n* (%)	19 (4.3)
SGA, *n* (%)	113 (25.4)
1 minute Apgar <7, *n* (%)	9 (2.0)
Neonatal death, *n* (%)	14 (3.1)

APO, adverse pregnancy outcome; pPROM, preterm premature rupture of membrane; PTB, preterm birth; PE, preeclampsia; SGA, small for gestational age.

### ROC analyses

We performed ROC analyses to determine the predictive thresholds of 24-hour proteinuria and serum C3 and C4 levels for at least one APO, PE, and PTB (Table 3).

**Table 3.** Cutoff Values of Complements and Proteinuria to Predict Adverse Maternal and Fetal Outcomes

	APOs			PE			PTB		
Values	Cutoff	Specificity	Sensitivity	Cutoff	Specificity	Sensitivity	Cutoff	Specificity	Sensitivity
24-hour urinary protein (g/L)	0.395	96.3%	28.2%	0.152	81.3%	67.7%	0.325	94.7%	36.6%
C3 (g/L)	0.64	90.9%	20.8%	0.64	88.7%	32.3%	0.64	90%	24.1%
C4 (g/L)	0.11	76.1%	39.6%	0.08	91.6%	33.8%	0.11	74%	42%

APOs, adverse pregnancy outcomes; PE, preeclampsia; PTB, preterm birth; C3, Complement 3; C4, Complement 4.

For predicting APOs, the ROC analyses revealed an optimal cutoff value of a 24-hour proteinuria level of 0.395 g/L, which had a sensitivity of 96.3% and a specificity of 28.2%. The optimal cutoff value for predicting PTB was a 24-hour proteinuria level of 0.325 g/L, which had a sensitivity of 94.7% and a specificity of 36.6%. Both of these cutoff values were higher than the lower limit of normal (>0.3 g/L). However, the cutoff value to predict PE was lower, at 0.152 g/L, with a sensitivity of 81.3% and a specificity of 67.7%.

The serum C3 level had the same optimal cutoff value of 0.64 g/L for predicting APOs, PE, and PTB, with sensitivities of 90.9%, 88.7%, and 90%, respectively, and specificities of 20.8%, 32.3%, and 24.1%.

The serum C4 level showed the best cutoff value for predicting APOs and PTB of 0.11 g/L, which had a sensitivity of 76.1% for APOs and 74% for PTB and a specificity of 39.6% for APOs and 42% for PTB. The cutoff for the serum C4 level to predict PE was slightly lower than the lower limit of normal, at 0.08 g/L, which had a sensitivity of 91.6% and a specificity of 33.8%.

### Predictors of at least one APO

Table 4 compiles the results of the univariate analysis. Women with at least one APO were more likely to be of advanced age (*p* = 0.018), to receive prenatal care at other centers (*p* < 0.001), to have associated APS (*p* < 0.001), and to have preexisting HTN (*p* = 0.006) than those without APOs. Furthermore, these patients were more prone to experience flare-ups during pregnancy (*p* < 0.001), were more likely to test positive for urinary sediment (*p* = 0.015), and had higher 24-hour proteinuria in the first trimester (*p* < 0.001). Both low C3 and low C4 were associated with APOs (*p* = 0.019 and *p* < 0.001, respectively). APO pregnancies more commonly involved immunosuppressive drugs (*p* = 0.003), whereas a smaller proportion of these pregnancies were exposed to HCQ (*p* = 0.003). Notably, there was no association between APO and LN or other previous SLE symptoms.

**Table 4.** Characteristics of Pregnant SLE Patients in the First Trimester, Pregnancy Outcomes, and Their Association with APOs, PE, and PTB

	At least one APO			PE			PTB		
Variables	At least one APO (*n* = 202)	No APO (*n* = 243)	*p*-value	PE (*n* = 65)	No PE (*n* = 380)	*p*-value	PTB (*n* = 145)	No PTB (*n* = 300)	*p*-value
Maternal characteristics									
Assisted pregnancy, *n* (%)	6 (3)	8 (3.3)	1	1 (1.5)	13 (3.4)	0.704	4 (2.8)	10 (3.3)	1
Age at conception, Median (Q1, Q3)	30 (27, 33)	31 (28, 34)	0.018^a^	30 (27, 34)	31 (28, 33)	0.864	30 (27, 33)	31 (28, 33)	0.02^a^
Regular follow-up, *n* (%)	117 (57.9)	228 (93.8)	<0.001^a^	23 (35.4)	322 (84.7)	<0.001^a^	73 (50.3)	272 (90.7)	<0.001^a^
SLE duration, years, Median (Q1, Q3)	5.96 (2, 9)	5 (3, 9.9)	0.231	6 (1, 8)	5 (2.92, 9.2)	0.349	5.54 (2, 9)	5.06 (3, 9.2)	0.127
Nulliparity, *n* (%)	170 (84.2)	196 (80.7)	0.402	52 (80)	314 (82.6)	0.736	118 (81.4)	248 (82.7)	0.841
Planned pregnancy, *n* (%)	152 (75.2)	226 (93)	0.082	46 (70.8)	332 (87.8)	1	101 (69.7)	277 (92.3)	0.006^a^
HTN, *n* (%)	15 (7.4)	4 (1.6)	0.006	12 (18.5)	7 (1.8)	<0.001^a^	11 (7.6)	8 (2.7)	0.031^a^
LN, *n* (%)	85 (42.1)	87 (35.8)	0.209	38 (58.5)	134 (35.3)	<0.001^a^	62 (42.8)	110 (36.7)	0.257
Associated APS, *n* (%)	30 (14.9)	12 (4.9)	<0.001^a^	19 (29.2)	23 (6.1)	<0.001^a^	28 (19.3)	14 (4.7)	<0.001^a^
History of PE, *n* (%)	11 (5.4)	5 (2.1)	0.098	6 (9.2)	10 (2.6)	0.019^a^	8 (5.5)	8 (2.7)	0.214
Previous thrombosis, *n* (%)	7 (3.5)	7 (2.9)	0.937	3 (4.6)	11 (2.9)	0.441	7 (4.8)	7 (2.3)	0.244
History of APOs, *n* (%)	55 (27.2)	66 (27.2)	1	21 (32.3)	100 (26.3)	0.394	40 (27.6)	81 (27)	0.987
Treatment during pregnancy									
LDA, *n* (%)	60 (29.7)	78 (32.1)	0.659	17 (26.2)	121 (31.8)	0.441	36 (24.8)	102 (34)	0.064
Prednisone, *n* (%)	164 (81.2)	186 (76.5)	0.283	50 (76.9)	300 (78.9)	0.838	116 (80)	234 (78)	0.719
LMWH, *n* (%)	22 (10.9)	28 (11.5)	0.953	9 (13.8)	41 (10.8)	0.611	16 (11)	34 (11.3)	1
HCQ, *n* (%)	149 (73.8)	208 (85.6)	0.003^a^	40 (61.5)	317 (83.4)	<0.001^a^	98 (67.6)	259 (86.3)	<0.001^a^
Immunosuppressive drug, *n* (%)	56 (27.7)	38 (15.6)	0.003^a^	23 (35.4)	71 (18.7)	0.004^a^	44 (30.3)	50 (16.7)	0.001^a^
Manifestations of SLE prepregnancy									
Rash, *n* (%)	85 (42.1)	104 (42.8)	0.955	22 (33.8)	167 (43.9)	0.166	60 (41.4)	129 (43)	0.824
Blood system, *n* (%)	69 (34.2)	87 (35.8)	0.793	26 (40)	130 (34.2)	0.445	58 (40)	98 (32.7)	0.157
Nervous system, *n* (%)	11 (5.4)	6 (2.5)	0.167	2 (3.1)	15 (3.9)	1	9 (6.2)	8 (2.7)	0.118
Arthritis, *n* (%)	78 (38.6)	83 (34.2)	0.381	22 (33.8)	139 (36.6)	0.776	55 (37.9)	106 (35.3)	0.668
Thrombocytopenia, *n* (%)	54 (26.7)	59 (24.3)	0.629	20 (30.8)	93 (24.5)	0.356	49 (33.8)	64 (21.3)	0.007
Urinary sediment, *n* (%)	75 (37.1)	63 (25.9)	0.015	36 (55.4)	102 (26.8)	<0.001	58 (40)	80 (26.7)	0.006
Laboratory characteristics in the first trimester									
24-h proteinuria, g/L, Median (Q1, Q3)	0.11 (0.09, 0.64)	0.1 (0.08, 0.12)	<0.001^a^	0.52 (0.12, 4.84)	0.1 (0.08, 0.13)	<0.001^a^	0.13 (0.1, 1.93)	0.1 (0.08, 0.12)	<0.001^a^
Positive anti-dsDNA antibodies, *n* (%)	65 (32.2)	73 (30)	0.702	28 (43.1)	110 (28.9)	0.033	43 (29.7)	95 (31.7)	0.748
Positive anti-SSA antibodies, *n* (%)	106 (52.5)	119 (49)	0.522	30 (46.2)	195 (51.3)	0.525	79 (54.5)	146 (48.7)	0.294
Positive anti-SSB antibodies, *n* (%)	21 (10.4)	36 (14.8)	0.213	4 (6.2)	53 (13.9)	0.124	17 (11.7)	40 (13.3)	0.745
At least one positive aPL, *n* (%)	53 (26.2)	51 (21)	0.234	23 (35.4)	81 (21.3)	0.02	40 (27.6)	64 (21.3)	0.18
Complement C3, Mean ± SD	0.85 ± 0.25	0.9 ± 0.22	0.019^a^	0.81 ± 0.26	0.89 ± 0.23	0.018^a^	0.84 ± 0.26	0.9 ± 0.22	0.013^a^
Complement C4, Median (Q1, Q3)	0.13 (0.1, 0.16)	0.15 (0.12, 0.18)	<0.001^a^	0.12 (0.06, 0.15)	0.14 (0.11, 0.18)	<0.001^a^	0.12 (0.09, 0.17)	0.14 (0.11, 0.18)	0.001^a^
Pregnancy outcomes									
Gestation weeks, Median (Q1, Q3)	35.2 (30.4, 37.2)	38.3 (38, 38.6)	<0.001^a^	31.2 (28.4, 36.2)	38.1 (37, 38.5)	<0.001^a^	33 (28.5, 35.3)	38.3 (37.9, 38.6)	<0.001^a^
Cesarean section, *n* (%)	139 (68.8)	165 (67.9)	0.918	50 (76.9)	254 (66.8)	0.142	100 (69)	204 (68)	0.923
Disease flare-ups, *n* (%)	81 (40.1)	24 (9.9)	<0.001^a^	34 (52.3)	71 (18.7)	<0.001^a^	73 (50.3)	32 (10.7)	<0.001^a^
Birth weight, g, Median (Q1, Q3)	2290 (1600, 2620)	3050 (2850, 3270)	<0.001^a^	1670 (1080, 2450)	2880 (2570, 3180)	<0.001^a^	1990 (1417.5, 2445)	2970 (2730, 3232.5)	<0.001^a^

^a^ *p* < 0.05.

LN, lupus nephritis; HTN, hypertension; PE, preeclampsia; APOs, adverse pregnancy outcomes; PTB, preterm birth; SLE, systemic lupus erythematosus; APS, antiphospholipid syndrome; LDA, low-dose aspirin; LMWH, low molecular weight heparin; HCQ, hydroxychloroquine; Immunosuppressive drugs, such as azathioprine (AZA), tacrolimus (TAC), and intravenous immunoglobulin (IVIG); anti-dsDNA: anti-double stranded DNA; aPL, antiphospholipid; SGA, small for gestational age.

The multivariate analysis focused on risk factors for APOs (Table 5), revealing that disease flare-ups (aOR 1.943; 95% CI, 1.026 to 3.677; *p* = 0.041), HTN (aOR, 5.790; 95% CI, 1.781 to 18.821; *p* = 0.004), and proteinuria in the first trimester (aOR, 3.687; 95% CI, 1.583 to 8.587; *p* = 0.002) were associated with the development of at least one APO. Regular follow-up at our center (aOR, 0.165; 95% CI, 0.085 to 0.319; *p* < 0.001) lowered the risk of APOs.

**Table 5.** Stepwise Regression Analysis of Risk Factors for Adverse Maternal and Fetal Outcomes in SLE Patients

Characteristics	Adjusted or (95% CI)	*p-*value
At least one APO		
Regular follow-up	0.165 (0.085–0.319)	<0.001
Disease flare-ups	1.943 (1.026–3.677)	0.041
HTN	5.790 (1.781–18.821)	0.004
24-hour proteinuria	3.687 (1.583–8.587)	0.002
PE		
Regular follow-up	0.177 (0.084–0.371)	<0.001
HTN	27.481 (7.928–95.256)	<0.001
History of PE	5.415 (1.396–21.002)	0.015
24-hour proteinuria	5.027 (2.428–10.407)	<0.001
C4	2.711 (1.174–6.261)	0.02
Associated APS	3.962 (1.558–10.072)	0.004
PTB		
Regular follow-up	0.259 (0.141–0.477)	<0.001
Disease flare-ups	2.841 (1.511–5.340)	0.001
HTN	3.579 (1.215–10.545)	0.021
HCQ use	0.544 (0.300–0.989)	0.046
24-hour proteinuria	2.835 (1.341–5.995)	0.006
Associated APS	3.530 (1.551–8.033)	0.003

APOs, adverse pregnancy outcomes; PE, preeclampsia; PTB, preterm birth; HTN, hypertension; APS, antiphospholipid syndrome; HCQ, hydroxychloroquine.

### Predictors of PE

Pregnancies complicated by PE showed higher rates of prenatal care at other centers, disease flare-ups, LN, HTN, associated APS, a history of PE, proteinuria, positive anti-dsDNA antibodies, at least one positive aPL, and lower serum C3 and C4 levels than those without PE (*p* < 0.001, *p* < 0.001, *p* < 0.001, *p* < 0.001, *p* < 0.001, *p* = 0.019, *p* < 0.001, *p* = 0.033, *p* = 0.02, *p* = 0.018, and *p* < 0.001, respectively). These pregnancies also had a higher proportion of patients using immunosuppressive drugs (*p* = 0.004) but fewer taking HCQ (*p* < 0.001). No significant association was found between PE and any SLE manifestation (Table 4).

The multivariate analysis identified HTN (aOR, 27.481; 95% CI, 7.928 to 95.256; *p* < 0.001), a history of PE (aOR, 5.415; 95% CI, 1.396 to 21.002; *p* = 0.015), associated APS (aOR, 3.962; 95% CI, 1.558 to 10.072; *p* = 0.004), proteinuria in the first trimester (aOR, 5.027; 95% CI, 2.428 to 10.407; *p* < 0.001), and low serum C4 in the first trimester (aOR, 2.711; 95% CI, 1.174 to 6.261; *p* = 0.02) as independent risk factors for PE. Regular follow-up at our center (aOR, 0.177; 95% CI, 0.084 to 0.371; *p* < 0.001) appeared to protect against PE (Table 5).

### Predictors of PTB

PTB was more likely with advanced age (*p* = 0.02), prenatal care at other centers (*p* < 0.001), unplanned pregnancies (*p* = 0.006), disease flare-ups (*p* < 0.001), an associated APS (*p* < 0.001), HTN (*p* = 0.031), proteinuria (*p* < 0.001), and low complement levels in the first trimester (*p* = 0.013 and *p* = 0.001, respectively), according to univariate analysis. Women who developed PTB were more likely to have a low platelet count (*p* = 0.007) and to use immunosuppressive drugs (*p* = 0.001). Those continuing to use HCQ were less likely to develop PTB (*p* < 0.001) (Table 4).

The multivariate analysis concluded that disease flare-ups (aOR, 2.841; 95% CI, 1.511 to 5.340; *p* = 0.001), HTN (aOR, 3.579; 95% CI, 1.215 to 10.545; *p* = 0.021), associated APS (aOR, 3.530; 95% CI, 1.551 to 8.033; *p* = 0.003), and proteinuria in the first trimester (aOR, 2.835; 95% CI, 1.341 to 5.995; *p* = 0.006) were independent risk factors for PTB. Regular follow-up at our center (aOR, 0.259; 95% CI, 0.141 to 0.477; *p* < 0.001) and the use of HCQ during pregnancy (aOR, 0.544; 95% CI, 0.300 to 0.989; *p* = 0.046) were linked with a lower risk of developing PTB (Table 5).

## Discussion

Our study primarily focused on the rates of APOs, specifically PE and PTB, in pregnancies complicated by SLE. Consistent with prior research,^21–26^ we found that 45.4% of SLE pregnancies experienced at least one APO, including 14.6% PE and 32.6% PTB. We also found that more than a quarter of newborns were classified as SGA and more than 60% of them were born preterm. Given our status as a tertiary referral hospital, many patients were referred to us after a diagnosis of severe SLE during pregnancy. This limited our ability to provide preconception counseling and timely treatment, resulting in an 85% incidence rate of at least one APO in this population. Nearly 33.9% of pregnancies still experienced at least one APO despite regular follow-up at our center. In our comprehensive analysis, disease flare-ups, HTN, and 24-hour proteinuria emerged as strong predictors for APOs, PE, and PTB. Remarkably, regular follow-ups reduced the risk of adverse maternal and fetal outcomes by 74%–84%.

Our findings showed that 23.6% of pregnancies were complicated by maternal lupus flares, aligning with previous reports.^21,24,25^ Persistent SLE activity or flares have been associated with more maternal and fetal complications.^8^ Our findings also identified disease flares as independent contributors to at least one APO and PTB risk. Among 46 newly diagnosed cases of SLE during pregnancy, 37 (80.4%) experienced at least one APO. PE occurred in 16 (34.8%) cases, while PTB occurred in 32 (69.6%) cases. Our study suggests the necessity of screening for SLE in women of childbearing age with suspected autoimmune diseases to enable early diagnosis, timely treatment, preconception counseling, and reduction of the risk of adverse maternal and fetal outcomes. Disease flare-ups could be considered as kinds of outcomes of pregnancies, while they can also be predictors of APOs along with other first trimester data, emphasizing the importance of monitoring disease activity during pregnancy.

Compared with the general population’s PE incidence of 3%–5%, SLE patients have a PE incidence ranging from 8% to 35%.^7,23,27–29^ Several studies have indicated a connection between LN, HTN, thrombocytopenia, hypocomplementemia, disease flare-ups, or the presence of aPLs and the development of PE.^30–32^ We identified HTN, a history of PE, associated APS, 24-hour proteinuria, and low serum C4 as independent risk factors for PE. Interestingly, disease flare-ups increased the risk of PE, albeit nonsignificantly in multivariable analysis. LN is associated with PE and other APOs.^21,33^ In a retrospective study involving 83 SLE patients with a total of 107 pregnancies, women with a previous diagnosis of LN had higher rates of PTB (30% vs. 11%) and PE (28% vs. 16%) than women without LN.^34^ However, LN did not seem to have an important impact on the development of these complications in our study. In this study, the diagnosis of LN relied not only on histopathological examination, as some patients did not undergo a renal biopsy. Instead, we based the LN diagnosis on results from 24-hour proteinuria or urine sediment analysis. This approach implies that certain patients might have had less severe manifestations of the condition. In addition, distinguishing between a recurrence of LN during pregnancy and preeclampsia symptoms can prove challenging, potentially leading to misdiagnosis or underdiagnosis.

Our results also showed that HCQ use during pregnancy reduced the risk of PTB. Its anti-inflammatory, immunomodulatory, antithrombotic, and vasculoprotective mechanisms potentially improve maternal–fetal outcomes.^35–37^ Retrospective studies found that the use of aspirin and HCQ had a protective effect against fetal loss and PE in patients diagnosed with SLE.^38,39^ However, we were unable to establish the efficacy of low-dose aspirin (LDA) and other treatments. As it has hardly any significant adverse effects on the fetus, according to current studies, it is highly recommended that HCQ and LDA should be used in all pregnant women complicated with SLE, although future studies are needed to elucidate the specific therapeutic mechanisms to understand the pathophysiological mechanisms of PE, PTB, and other APOs.

Our study emphasizes the importance of proteinuria screening in early pregnancy, even in the absence of LN, given its significant association with APO rates. In addition, low complement levels in the first trimester could predict APO, PE, and PTB risks. Notably, C4 level in early pregnancy seemed to be a reliable predictor of PE, although serum C3 and C4 cutoff values were significantly below the lower limit of the normal range.

Generally, urine protein levels exceeding 0.3 g per day at any point during pregnancy are considered abnormal. Few studies have calculated specific cutoff values for 24-hour proteinuria that could indicate the risk for at least one APO, PE, or PTB.^8^ Our data suggest that proteinuria exceeding 0.395 g/day in the first trimester can significantly increase the risk of at least one APO. Furthermore, PTB rates escalated when 24-hour proteinuria exceeded 0.325 g—a value near the lower threshold of the normal range. Notably, the likelihood of developing PE increased significantly when 24-hour proteinuria was over 0.152 g, which was well below the lower limit of the normal range. The finding underscored the importance of early pregnancy proteinuria screening, even in the absence of LN.

Consistent with previous studies, our study indicated that lower complement levels in the first trimester may reflect heightened complement activation. This condition leads to anaphylatoxin generation, contributing to deficient placental vascularization and trophoblast injury.^40^ Low serum C3 levels and high anti-DNA antibody titers at conception have been linked to poor pregnancy outcomes, such as fetal death and PTB, in SLE pregnancies.^28,40^ Our study suggests that serum C3 below 0.63 g/L in the first trimester can significantly increase the risk of at least one APO, PE, and PTB. Similarly, serum C4 below 0.11 g/L pose a risk for at least one APO and PTB, although these associations were not statistically significant in multivariable analysis. Strikingly, the increased incidence of PE in pregnancies with low serum C4 (<0.08 g/L) implied that early pregnancy C4 level could be a reliable PE predictor. We found that the cutoff values for serum C3 and C4 (0.73 g/L and 0.1 g/L, respectively, at our center) were significantly below the lower threshold of the normal range.

Discrepant findings between studies can result from factors such as patient selection, study design, use of control groups, term definitions, SLE heterogeneity, and difficulties distinguishing pregnancy features (*e.g.,* proteinuria, thrombocytopenia) from active SLE manifestations. Consequently, identifying appropriate strategies to handle the unique needs and risks associated with SLE pregnancies requires thoughtful consideration. Our retrospective study at a tertiary-care referral center explored pregnancies complicated by SLE. The study’s strengths included its relatively large sample size and its ability to better guide personalized obstetric monitoring of SLE pregnancies. Its main limitations are its single-center retrospective design and its lack of information for some analyses due to data unavailability. As patients may not have been referred to our center for further evaluation after an early pregnancy loss and the reasons for early pregnancy loss are complicated, we did not include fetal loss in our study. While the incidence of other adverse fetal outcomes, such as stillbirth, neonatal death, and SGA, was higher in SLE pregnancies (32.8% in total), further research is needed to investigate their associated risk factors and predictive factors.

Despite these limitations, this study provides essential insights into obstetric monitoring of SLE pregnancies, emphasizing the need for adequate preconception counseling, continuing HCQ usage during pregnancy, and regular antenatal follow-up by experienced obstetricians and rheumatologists.

## Abbreviations Used

aCL: anticardiolipin
anti-dsDNA: anti-double stranded deoxyribonucleic acid;
anti-β2GPI: anti-Beta2 glycoprotein type I
aPLs: antiphospholipid antibodies
APOs: adverse pregnancy outcomes
APS: antiphospholipid syndrome
AZA: azathioprine
C3: complement 3
C4: complement 4
DM: diabetes mellitus
DORIS: Definition of Remission in SLE
HCQ: hydroxychloroquine
HTN: hypertension
IQRs: interquartile ranges
IVIG: intravenous immunoglobulin
LA: lupus anticoagulant
LDA: low-dose aspirin
LMWH: low molecular weight heparin
LN: lupus nephritis
PAH: pulmonary arterial hypertension
PE: preeclampsia
pPROM: preterm premature rupture of membrane
PTB: preterm birth
ROC: receiver operating characteristic
SDs: standard deviations
SGA: small for gestational age
SLE: systemic lupus erythematosus
SLEDAI-2K: SLE Disease Activity Index-2000
TAC: tacrolimus
